# Progression of Repair and Injury in Human Liver Slices

**DOI:** 10.3390/ijms19124130

**Published:** 2018-12-19

**Authors:** Alison E. M. Vickers, Anatoly V. Ulyanov, Robyn L. Fisher

**Affiliations:** 1 Human Translational Models LLC, Irvine, CA 92612, USA; 2 Inova Translational Medicine Institute, Inova Hospital, Fairfax, VA 22031, USA; anatoly@ulyanov.net; 3 Vitron, Inc., Tucson, AZ 85747, USA; robyn@vitron.com

**Keywords:** repair and injury, human liver slices

## Abstract

Human liver slice function was stressed by daily dosing of acetaminophen (APAP) or diclofenac (DCF) to investigate injury and repair. Initially, untreated human liver and kidney slices were evaluated with the global human U133A array to assess the extended culture conditions. Then, drug induced injury and signals of repair in human liver slices exposed to APAP or DCF (1 mM) were evaluated via specific gene expression arrays. In culture, the untreated human liver and kidney slices remained differentiated and gene expression indicated that repair pathways were activated in both tissues. Morphologically the human liver slices exhibited evidence of repair and regeneration, while kidney slices did not. APAP and DCF exposure caused a direct multi-factorial response. APAP and DCF induced gene expression changes in transporters, oxidative stress and mitochondria energy. DCF caused a greater effect on heat shock and endoplasmic reticulum (ER) stress gene expression. Concerning wound repair, APAP caused a mild repression of gene expression; DCF suppressed the expression of matrix collagen genes, the remodeling metalloproteases, cell adhesion integrins, indicating a greater hinderance to wound repair than APAP. Thus, human liver slices are a relevant model to investigate the mechanisms of drug-induced injury and repair.

## 1. Introduction

Pathways of organ repair and drug-induced injury are investigated in the 3-dimensional organotypic ex vivo model of human liver slices. Tissue architecture and organ function are maintained to mimic in vivo function. Advances in tissue processing and the daily exchange of medium have extended culture times. Additionally, the daily dosing of drugs has allowed for mechanistic forecasts of liver injury underlying clinical changes [1,2].

Drug-induced liver injury usually includes the culmination of oxidative stress, dysfunction of proteins in the endoplasmic reticulum (ER), a loss of mitochondria energy, resulting in apoptosis, necrosis and inflammation [3,4,5,6,7,8]. Contributing to these mechanisms may include the formation of reactive drug intermediates by hepatocytes or even peroxidases in Kupffer cells [9,10,11,12,13]. Liver slices generate the spectrum of drug metabolites as detected in vivo [14,15]. With on-going injury, tissue response may trigger pathways of repair and regeneration [16]. Endothelial and Kupffer cells contribute to an inflammatory response which could stimulate repair, while hepatic stellate cells are the major mediators of matrix formation in response to injury [17,18].

Organ slices have two cut surfaces, and with the extension of human liver slice viability for days in culture, both parameters could trigger repair processes. Evidence of tissue repair in untreated human liver slices is compared to human kidney slices in this study. Additionally, drug-induced injury and repair in human liver slices following acetaminophen (APAP) and diclofenac (DCF) exposure are investigated in this study. Both drugs cause liver dysfunction, marked by increases in serum transaminases, in some individuals. The underlying mechanism likely involves oxidative stress, induced by reactive metabolite formation, which impacts liver glutathione (GSH) status and mitochondria function [1,15,19,20]. APAP blood levels of 1 mM are associated with altered liver function in healthy volunteers, while 0.5–2 mM is associated with a high acute toxicity and severe hepatotoxicity [20,21,22]. DCF peak plasma therapeutic concentrations can reach 10 µM, while toxic plasma concentrations have been reported to reach 203 µM [20,23]. Hence, the daily dosing used in this study was intended to stress the coping mechanisms of a drug induced-cumulative effect of oxidative stress to reveal the resultant consequences, and to determine if repair processes were in effect. Gene expression changes indicative of organ dysfunction and changes in repair pathways demonstrate the potential of human liver slices to characterize drug injury and repair.

## 2. Results

### 2.1. Evidence of Regeneration

**Figure 1:**
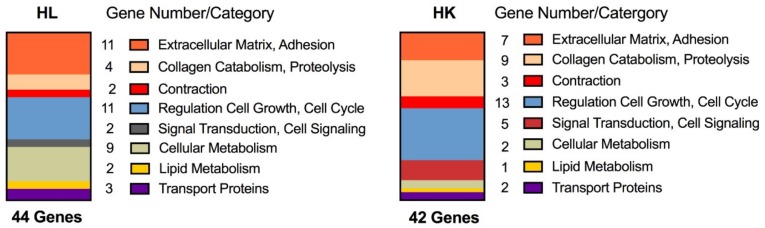
Functional gene categories and the mean percentage of significant gene expression changes induced in human liver slices (HL714, four slices/time point) and human kidney slices (HK3, four slices/time point). Gene expression changes were detected using the human U133A Affymetrix genome array, and days 2–4 are compared to day 1. The number of genes represented by each category is based on the total number of significant gene changes for each tissue.

**Figure 2:**
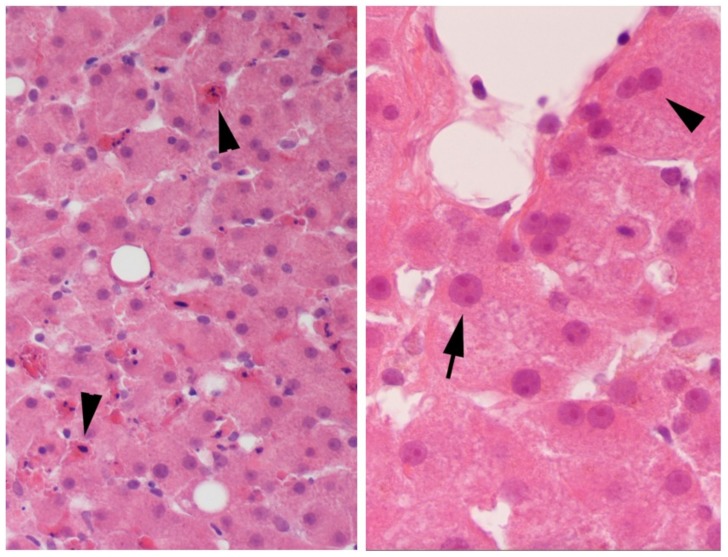
Evidence of liver regeneration is shown with arrows by the variation in nuclei size, anisokaryosis (**left panel**), and scattered multinucleated hepatocytes (**right panel**) in untreated human liver slices incubated for 3 days with daily exchange of medium. The magnification was 400×.

Both human liver slices and human kidney slices, cultured for several days with the medium changed daily, exhibited gene expression changes indicative of repair. Global gene expression profiling was performed using the human U133A Affymetrix gene chip array on human liver (HL714) slices or human kidney (HK3) slices cultured for 2–4 days (4 slices/timepoint) and compared to day 1. For both tissues, a small number of genes were significantly altered, 44 in human liver slices and 42 in human kidney slices, which represented similar structural or functional categories (Figure 1). The gene expression changes which occurred on day 2 persisted throughout the culture period for each tissue. Several extra-cellular matrix collagens were up-regulated in liver and kidney slices. Additionally, in the liver slices cell adhesion genes, laminins, were up-regulated. In contrast to the liver, the kidney slices exhibited an up-regulation of the collagen catabolism matrix metalloproteinases, *MMP* 1-3, 7, 12, and 14 (Table 1). Several growth factors were up-regulated, particularly in the human liver slices, including epidermal growth factor (*EGF*), *epiregulin*, and the proteolytic cascade *IGFBP-5*; while *TGF-β1* was up-regulated in liver and human kidney slices. Kidney slices exhibited an up-regulation of markers of DNA synthesis and cell cycle genes including mini-chromosome maintenance (*MCM*) and the microtubule associated kinesins, as well as an up-regulation of cytokine *IL-24* which is linked with wound repair and cell survival. In spite of the gene expression profiles indicative of repair in both tissues, the morphology revealed an improvement in liver slice viability in contrast to the kidney. By day 3 of culture, the human liver slices displayed a variation in the size of nuclei (anisokaryosis) and scattered multinucleated hepatocytes that continued through day 6, both indications of regeneration (Figure 2), with no evidence of progressive necrosis or fibrosis. Bile production was visible in the liver slices over the culture time. However, the human kidney slices exhibited progressive necrosis, in spite of gene changes indicative of repair.

### 2.2. APAP and DCF Toxicity Profiling

Human liver slices, exposed to daily dosing of APAP or DCF, were evaluated with specific gene expression arrays to reveal the direct effects of each drug on the liver. Daily dosing was selected to mimic a clinical regime with multiple exposures. Key genes of pathways activated in response to injury are represented on the Human Molecular Toxicology PathwayFinder RT^2^ Profiler^TM^ PCR array (370 genes); genes involved in the wound healing response were queried with the Human Wound Healing RT^2^ Profiler PCR array (84 genes).

**Figure 3:**
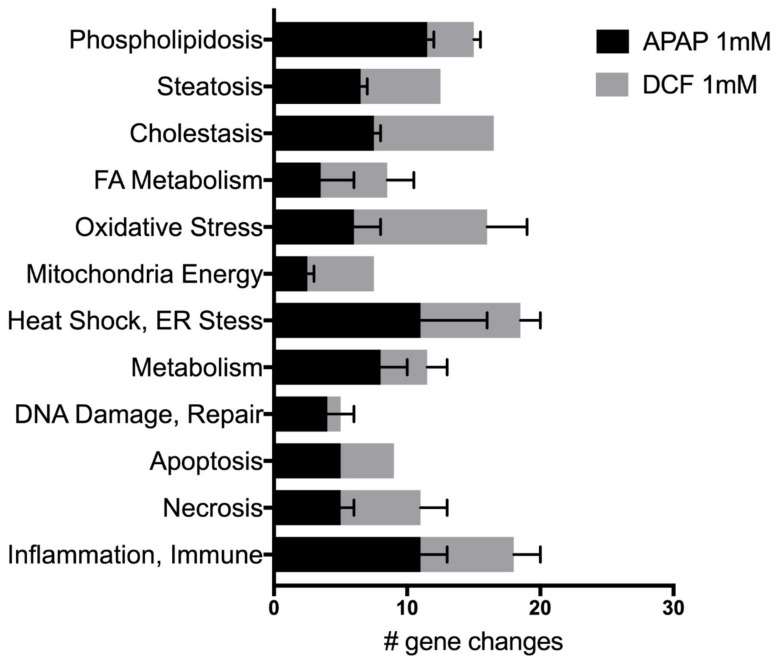
Potential side-effects categorized by the number of significant gene expression changes induced by acetaminophen (APAP) 1 mM (acetaminophen) or by diclofenac (DCF) 1 mM (diclofenac) after 72 h of exposure. Values represent the mean ± SEM of significant gene expression changes in human liver slices from HL870 and HL871 (10 control and 6 treated slices/liver) from the Human Molecular Toxicology PathwayFinder RT^2^ PCR array.

Genes indicative of several potential liver toxicities revealed the direct and multi-factorial effects of APAP and DCF exposure to human liver slices following three daily doses. APAP (1 mM) significantly altered up to 21% of the 370 genes on the Molecular Toxicology array, while DCF significantly altered up to 24% of the total genes queried. Both drugs altered the expression of genes associated with cholestasis, steatosis and phospholipidosis, as well as genes associated with the underlying mechanisms, altered fatty acid metabolism, oxidative stress, changes in mitochondrial energy, ER stress and heat shock response, which can affect the genes indicative of DNA damage and repair, apoptosis, necrosis and the inflammation and immune response (Figure 3).

Significant gene changes induced by APAP with a false discovery rate (FDR) < 15% represented up to 41% of the significant gene changes (63 genes in HL870 and 77 genes in HL871) of just a few categories (Table 2). Several of the same genes were altered by both human livers. Changes in the expression of genes indicative of cholestasis represented changes in the transporters *ABCC2* and *UGT2B4*, which are up-regulated by the farnesoid X receptor and are involved in the glucuronidation of the bile acid hyodeoxycholic acid. Additionally, they function as a regulator of cholesterol or fatty acid biosynthesis (*SREBF1*, *SCD*, *CPT2*), and in cholesterol conversion (*CYP7A1*). Oxidative stress gene expression changes included the *DUOXs* associated with reactive oxygen species formation, *GPXs* linked with the detoxification of hydrogen peroxide, *PRDX* antioxidant activity and *NQO1* which encodes for NADPH dehydrogenase (quinone 1) and is involved in the reduction of quinones. In contrast, DCF exposure altered the expression of two genes *MKI67* and *LYZ* at the FDR < 15% level.

Significant gene changes induced by APAP with an FDR < 30% represented several categories linked with organ toxicity. Gene changes indicative of cholestasis were transporters. Additional categories included fatty acid metabolism (*CPT1B*), mitochondria energy (*CYC1*, *IDHs*, *MDH1B*), heat shock (*HSPAs, DNAJs*) oxidative stress (*PRDXs*), apoptosis (*BCLs*), necrosis (*BMF3*), as well as inflammation and immunotoxicity (*IL-6*, *C9*).

Significant gene changes induced by DCF with an FDR < 30% represented 20–24% of the 370 genes queried and were indicative of several toxicity categories. DCF down-regulated many genes indicative of phospholipidosis, steatosis and cholestasis except for the *ABCB1*, which encodes for p-glycoprotein to aid in the elimination of xenobiotics, and WIPI1, which interacts with phospholipids. Oxidative stress genes were up-regulated by DCF and included genes indicative of antioxidant activity, *PRDX6* and *GPXs*; as well as *POR,* which encodes for P450 reductase, and *PPP1R15B*, which is linked with translation. A mitochondria energy gene *COX6B1*, which encodes a cytochrome c oxidase, was up-regulated. Numerous heat shock (*DNAJs*, *HSF2*, *HSPAs*) and ER stress (*ATFs*, *EIF2AK3*) genes were up-regulated. Several DNA damage and repair genes were also up-regulated (*DDIT3*, *ERCCs*, *MDM2*, *XRCC1*). Genes indicative of apoptosis (*GADD45*, *TNFSF 10A* and *B*, *TP53*) and necrosis (*CYCLD*, *PARP2*, *TXNL4B*) were up-regulated. Most of the genes indicative of inflammation and immunotoxicity were down-regulated.

A comparison to 48 h gene expression data from the same two livers and analyzed via the Molecular Toxicology array revealed a similar pattern in the proportion of significant genes altered for each toxicity category. The number of significant gene changes increased from 48 h to 72 h: 1.4–2.5-fold for APAP and 1.5–2.1-fold for DCF (Table 3). The toxicity categories that emerged across the human livers and the two time-points with the highest proportion of gene expression changes for APAP were cholestasis, steatosis and phospholipidosis (29–33%), necrosis (6–15%) plus inflammation and immunotoxicity (8–12%). Underlying mechanisms included heat shock and ER stress (12–15%), oxidative stress (11–14%), mitochondria energy (5–10%), fatty acid metabolism (6–9%) and apoptosis (5–10%). Similar to APAP, the toxicity categories induced by DCF exhibiting the highest proportion of significant gene expression changes included cholestasis, steatosis and phospholipidosis (20–34%) plus inflammation and immunotoxicity (9–14%). Key differences are that DCF induced more apoptosis (6–15%) and less necrosis (5–6%), and a greater extent of heat shock and ER stress (18–27%) than APAP. The underlying mechanisms for DCF also include oxidative stress (6–9%), mitochondria energy (2–4%) and changes in fatty acid metabolism (7–8%).

### 2.3. Functional Assays

**Figure 4:**
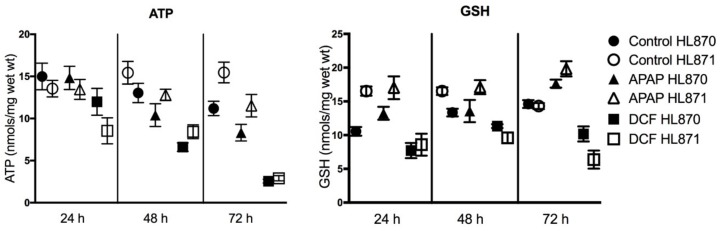
Adenosine triphosphate (ATP) and glutathione (GSH) levels (24–72 h) in human liver slices exposed to daily dosing of APAP (1 mM) or DCF (1 mM). Values (nmols/mg wet weight (wt)) are presented as the mean ± SEM from 10 control slices or six treated slices/time-point from two human livers (HL870, HL871) and related to the time-matched control value.

Selected functional assays aid in defining the potential consequences and risks of forecasted side-effects from gene expression profiling data. The functional endpoints of adenosine triphosphate (ATP) and glutathione (GSH) levels address the consequences of oxidative stress including changes in GSH status and changes in mitochondria energy. Human liver slices synthesize and maintain both ATP and GSH levels with time in culture over 72 h. APAP exposure caused significant decreases in human liver slice ATP levels, 20% at 48 h and 25% at 72 h. DCF also caused significant decreases of ATP levels, 47% at 48 h, and 80% by 72 h, indicative of limited ATP synthesis and decreased mitochondria energy, which did not affect RNA yield or integrity. GSH synthesis was stimulated by APAP exposure with GSH levels significantly increased 30% at 72 h. DCF exposure significantly decreased GSH levels, 37% at 24 h, which rebounded, then represented a 43% decline at 72 h (Figure 4). An increase of GSH levels as seen with APAP is indicative of the liver tissue coping with oxidative stress, while the decline of GSH levels by DCF demonstrates the inability of the liver tissue to cope with the stress. Biomarkers of GSH status and mitochondria energy could benefit identification of adverse liver effects and therapy.

### 2.4. Wound Repair

**Figure 5:**
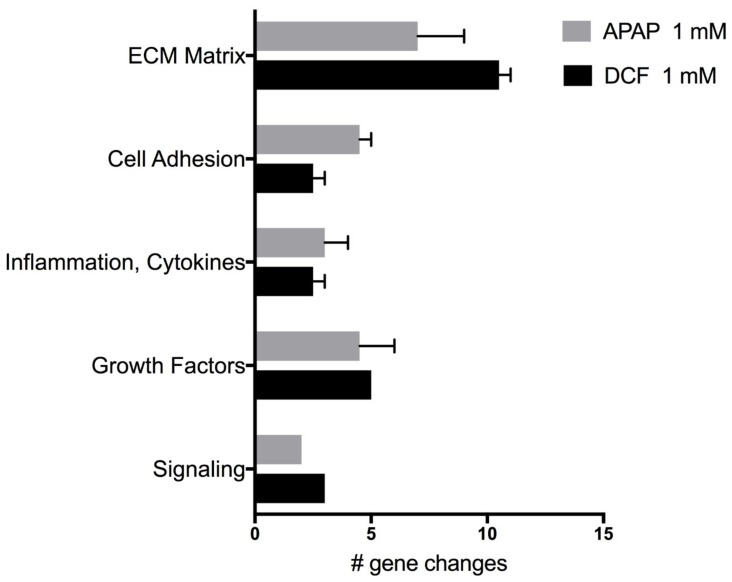
Wound repair categories and number of significant gene expression changes induced by APAP 1 mM (acetaminophen) or by DCF 1 mM (diclofenac) after 72 h of exposure. Values represent the mean ± SEM of significant gene expression changes in human liver slices HL870 and HL871 (10 control and six treated slices/liver) from the Human Wound Healing RT^2^ PCR array.

The expression of genes linked with wound repair were evaluated following APAP (1 mM) or DCF (1 mM) exposure for 72 h, a time-point where human liver slices exhibit regeneration (Figure 5). Each drug triggered a different response in the same human livers (HL870, HL871). Overall, APAP caused mild repression of several genes in comparison to DCF, which caused severe repression of many genes (Table 4). DCF greatly suppressed extra-cellular matrix (ECM) collagen genes, ECM remodeling genes like matrix metalloproteases (MMPs), cell adhesion integrin genes and *WISP1*, a signaling pathway that attenuates apoptosis, suggesting a greater hindrance to wound repair for DCF than for APAP. An up-regulation of genes by DCF included *MMP-1*, a remodeling gene of interstitial collagenase and fibroblast collagenase, the fibroblast growth factor (*FGF2*), and cytokine (*CSF3*). The up-regulation of the PTGS2 gene, which encodes for cyclooxygenase-2 (*COX2*), is a known pharmacologic target of DCF to suppress inflammation, and likely reflects a compensation response from the inhibitory action of DCF.

## 3. Discussion

This study evaluated tissue repair in untreated human liver slices and in the presence of drug-induced injury. Organ slices, with two cut surfaces from preparation, remain viable for several days in rotating cultures with daily medium exchange [24]. One part of this study was to evaluate recovery and repair processes, generated by optimizing the culture conditions, in untreated human liver slices compared to human kidney slices. Then, drug induced injury and repair were evaluated in human liver slices following exposure to APAP and DCF. Both drugs cause hepatoxicity clinically, as detected by increases in serum transaminases in some individuals. Additionally, both drugs form reactive metabolites which could induce oxidative stress, altering the GSH status, causing endoplasmic reticulum stress and affecting mitochondria function [2,15,22]. This ex vivo model of human liver slices, which mimics in vivo due to the presence of all the cell types, is a relevant model to provide insight into mechanisms of oxidative stress and the consequences of drug-induced injury to identify drug safety risks.

Both gene expression and morphology were used to evaluate the culture conditions of untreated human liver slices and human kidney slices. Previously reported is that repair was evident in untreated rat liver slices and human liver slices [25,26]. In this study, additional human liver data was shown and compared with human kidney slices. A broad look at gene expression was performed with the human genome array U133, only to yield a small number of significant gene changes. Both tissues exhibited gene expression profiles that indicated the activation of repair pathways. In particular, several collagens were up-regulated in both tissues. In human liver slices, the cell adhesion genes, laminins, and growth factors were up-regulated; in human kidney slices, several collagen catabolism matrix metalloproteinases were up-regulated. Additionally, in human kidney slices, cell cycle and markers of DNA synthesis were up-regulated, as well as cytokine IL-24, which is linked with cell survival. Morphology revealed regeneration in the human liver slices with the variation in the size of the nuclei (anisokaryosis) and scattered multinucleated hepatocytes, from days 3–6 of culture. Human kidney slices exhibited progressive necrosis in the proximal tubular epithelium from days 3–6. In humans the liver can regenerate back to its original mass and there is tissue orchestration of regeneration versus fibrosis [27,28]; the kidney can repair, yet it has limited regenerative capacity [29].

Drug induced injury and repair were investigated in human liver slices exposed to APAP (1 mM) or DCF (1 mM). The doses of the drugs for this study were selected from literature citing high plasma concentrations associated with hepatotoxicity and from previous rat and human liver slice studies exhibiting concentration and time response changes in tissue function [2,15,19,20,21,22,23,30]. Both drugs were tested side-by-side in each human liver, and the drugs were dosed daily to increase the overall stress on liver slice function. Genes linked to organ injury were interrogated following three doses of APAP or DCF and compared to time-matched control liver slice RNA with the Human Molecular Toxicology PathwayFinder RT^2^ PCR array (370 genes) and the Human Wound Healing RT^2^ PCR array (84 genes). The significant gene expression changes revealed multi-factorial and direct consequences of APAP or DCF exposure to human liver slices. Several targets of dysfunction and underlying mechanisms were identified. This study, with three days of dosing, stressed the human liver slices more than the previous two days of dosing, as evidence by the increased number of significant gene changes [30].

The categories of cholestasis, steatosis and phospholipidosis exhibited the highest percentage of significant gene expression changes for APAP and DCF; however, it is the effects on transporter genes that stands out and is indicative of cholestasis. Several liver transporter genes exhibited altered expression, in particular the efflux p-glycoprotein transporter ABCB1 (APAP, DCF), the organic anion transporters, ABCC2 and ABCC3 (APAP), the organic solute transporter SLC51A (DCF), and the sodium independent transporter SLCO1A2 (DCF). Genes associated with the conversion of cholesterol to bile acids, CYP7A1 was up-regulated by APAP, while CYP7A1 and CYP7B1 were down-regulated by DCF. The elimination of bile acids by UGT2B4 was up-regulated by APAP, and the UGT1A1 was down-regulated by DCF. The perturbation of several transporter genes by drugs and elimination pathways may lead to some congestion of bile acids in the tissue, while the tissue is trying to cope with the export of bile acids and solutes to prevent cholestatic hepatotoxicity. Drug-induced cholestasis and impairment of bile flow may be induced by APAP and DCF in some individuals with severe hepatotoxicity. The clinical manifestations are typically elevated transaminase levels, and in severe cases occur in conjunction with jaundice and lactic acidosis [22,31,32,33]. Moreover, both APAP and DCF affect some genes erratically associated with fatty acid metabolism, suggesting that the side-effects of steatosis and phospholipidosis are less likely to occur. Liver lipid deposits and lactic acidosis evident with hepatotoxicity can also be attributed to inhibition of the respiratory complexes of the electron chain in the mitochondria, decreased energy (ATP) production and oxidative stress [34].

APAP and DCF induced gene expression changes indicative of oxidative stress and mitochondria energy. In particular, the oxidative stress genes affected by both drugs included reactive intermediate formation (*DUOXs*, *GPXs*, *EPHX1*), regulators of redox status peroxiredoxin (*PRDXs*), while a regulator of translation (*PPP1R15B*) was up-regulated by DCF. Mitochondria energy genes affected by APAP included the matrix succinyl-CoA ligase (*SUCLG1*) gene, and the trichloroacetic acid (TCA) cycle gene *MDH1* (malate dehydrogenase). DCF altered an uncoupling protein (*UCP2*), which accelerates energy use through the separation of oxidative phosphorylation from ATP synthesis. Mechanistic studies suggest that the uncoupling of mitochondrial respiration by the uptake of DCF by the mitochondrial anion carrier results in opening the permeability transition pore to initiate cell death [35,36]. Other studies suggest that DCF reactive electrophiles bind to mitochondria proteins, which triggers apoptosis and possibly a hypersensitivity reaction [37,38,39].

APAP and DCF perturb tissue anti-oxidant status via different mechanisms. The resulting oxidative stress involves mitochondria injury, ER stress and effects GSH levels. For APAP, the direct conjugation of the N-acetyl-p-benzoquinone imine (NAPQI) metabolite with GSH, and formation of toxic metabolites by peroxidase-like enzymes can reduce the liver anti-oxidant status [35]. Diclofenac, a non-steroidal anti-inflammatory agent, forms reactive metabolites that can bind to cellular macromolecules and proteins, altering tissue function. Mitochondria are a key target for APAP and DCF. In this study, both APAP and DCF caused significant time-dependent decreases of ATP levels, which may reflect an inhibition of ATP synthesis, since other tissue functions like RNA yield and integrity remained intact. GSH levels were significantly decreased with DCF, while significant increases occurred with APAP. Since the metabolism of APAP consumes GSH via a metabolic pathway, the tissue responded by synthesizing GSH. Dose response studies in previous human liver slice studies, have reproducibly demonstrated that 1 mM APAP and DCF affects energy and antioxidant status, ATP or GSH levels, in most but not all human liver slice studies [2,15]. A reduction of tissue anti-oxidant status and mitochondria function triggers liver injury or necrotic cell death [3,39,40,41,42]. Liver GSH decreases can sensitize hepatocytes to the oxidative effects of cytokines such as tumor necrosis factor [43,44]. The FDA is requiring additional labeling for APAP [45]. Acetaminophen is the primary choice for pain in USA hospitals, which may not be the best choice, since the sick and elderly likely have a compromised liver anti-oxidant status.

An increase of unfolded proteins in the endoplasmic reticulum (ER) triggers a stress response, that if not stabilized, effects the accumulation of unfolded proteins and destruction of misfolded proteins, and can lead to cell death [5,43]. In this study, APAP induced the expression of the heat shock genes *HSP90AA1*, *HSPA1A* and *HSPA1B*, which code for proteins that act as chaperones to stabilize existing proteins and facilitate the folding of newly translated proteins. DCF up-regulated the expression of a mitochondrial heat shock gene (*HSP9A*) and the protein folding genes (*DNAJ* family). Additionally, DCF up-regulated more of the ER stress genes, including the gene encoding for the transport of unfolded proteins to the proteasome for degradation (*SERP1*), and genes that encode for heat shock transcription factors (*ATF4*, *ATF6*), and a gene coding for a translation-initiation factor (*EIF2AK3*).

DCF up-regulated the expression of several genes associated with apoptosis, including the growth arrest gene *GADD45*, and genes of the TSF-receptor family (*TNFRS10A* and *B*) involved with apoptosis, and a gene linked with excision repair of damaged DNA, *PARP2*. APAP suppressed the expression of *Bcl2*, *Bcl2L11*, and the Bcl-2 modifying factor, *BMF*. In a previous study, after 48 h, both APAP and DCF exposure up-regulated the genes for the death receptor FAS (*FAS*, *FADD*), while APAP up-regulated several genes linked with necrosis [42].

Interleukin genes (*IL1B*, *IL6*) and the cytokine gene TNF, as well as genes of molecules that influence the immune system (*C9*, *CD36*, *CD86*) were sporadically suppressed by DCF or APAP, which can be suggestive of an inflammation and possible immune response. It has been reported that proinflammatory and cytotoxic mediators released from macrophages, may contribute to APAP hepatotoxicity in some individuals [46].

The expression of wound repair genes following drug-induced injury by APAP (1 mM) or DCF (1 mM) was examined at a time-point (72 h) where untreated human liver slices begin to exhibit regeneration. Overall, APAP caused a mild repression of several genes in comparison to DCF which caused severe repression. DCF greatly suppressed extra-cellular matrix (ECM) collagen genes, ECM remodeling genes like matrix metalloproteases (MMPs), cell adhesion integrin genes suggesting a greater hinderance to wound repair for DCF than for APAP. Gene expression that was up-regulated by DCF in both livers included MMP-1, a remodeling gene also known as interstitial collagenase and fibroblast collagenase, fibroblast growth factor *FGF2*, the cytokine *CSF3*. The up-regulation of the *PTGS2* gene which encodes for cyclooxygenase-2 (*COX2*), is a known pharmacologic target of DCF to suppress inflammation. The up-regulation of *PTGS2* gene expression may reflect a compensation response from the inhibitory action of DCF. In a previous study, DCF delayed corneal wound healing via gene expression and by the rate of wound closure [47].

## 4. Materials and Methods

### 4.1. Chemicals and Reagents

Acetaminophen (cat # A7085) and diclofenac sodium salt (cat # PHR1144) were purchased from Sigma-Aldrich (St. Louis, MO, USA). The V-7 preservation solution was supplied by Vitron (Tucson, AZ, USA) [32]. Waymouth’s MB 752/1 (without L-glutamine, phenol red and sodium bicarbonate) culture medium and fetal bovine serum were purchased from Invitrogen (Chicago, IL, USA); while L-glutamine, Antibiotic/Antimycotic solution (100×) and gentamicin sulfate were obtained from Sigma-Aldrich (St. Louis, MO, USA). Mixed cellulose-ester IMMOBILIN-NC filters (HATF, 0.45 µm surfactant and triton free, autoclavable), used to support the liver slices on top of the rollers, were obtained from Millipore (Bedford, MA, USA). Dithiobis-nitrobenzoic acid, reduced GSH luciferin, luciferase and ATP were purchased from Sigma-Aldrich (St. Louis, MO, USA).

### 4.2. Biologicals

Human liver and kidney tissue was procured with donor consent using procedures approved by The International Institute for the Advancement of Medicine (IIAM, Edison, NJ, USA) Review Committee (DC-60508/0045-2213889v1, 18 April 2006). Extensive serologies demonstrated absence of infectious agents including HIV and hepatitis. The organ was perfused with Belzer UW Cold Storage Solution (Bridge To Life Ltd, Columbia, SC, USA) by the hospital following protocols for organ transplantation. Reasons for the availability of organs for research included anatomical irregularities, histological findings, age of donor, time in transit or status of the recipient. The organs used in this study were of transplantation grade and transported to the Vitron laboratory (Tucson, AZ, USA) in Belzer UW Cold Storage Solution on ice. Donor information and tissue viability is listed in Table 5. Tissue viability was checked in the initial slices prepared and at 24 h. Slice potassium (K^+^) values > 70 µmols/g wet weight is a marker for highly viable slices, linked with good ATP values, high RNA integrity and yield and stable morphology.

### 4.3. Human Liver Slice Cultures

The human liver and kidney tissue was cored (8 mm diameter) and kept mosit with V-7 cold preservation solution (Vitron Inc., Tucson, AZ, USA). Precision-cut slices (200 ± 25 µm thick) were cut in oxygenated V-7 cold preservation solution using the Brendel/Vitron tissue slicer (Vitron Inc, Tucson, AZ, USA). Each slice is supported on a sterile Immobilon-NC transfer membrane, placed on a titanium roller insert (Vitron Inc, Tucson, AZ, USA), and maintained in a 20 mL glass scintillation vial containing 1.7 mL of Waymouth’s MB 752/1 tissue culture medium, fortified with 2.24 g/L sodium bicarbonate, 0.35 g/L l-glutamine, 10 mL/L Antibiotic/Antimycotic solution, 84 µg/mL gentamicin sulfate and 10% fetal bovine serum. The human liver and kidney slice cultures were incubated in a Dynamic Organ Culture Incubator (Vitron Inc, Tucson, AZ, USA), rotating at 1 rpm and maintained at 37 °C in a 95% O_2_:5% CO_2_ atmosphere.

Human liver (HL714) and kidney slices (HK3) were maintained for several days in culture to extend slice viability in culture and to compare the gene expression changes with time in culture. The medium was exchanged every 24 h for up to 6 days with human liver slices and for human kidney slices. Every 24 h liver or kidney slices were collected (four slices/timepoint/assay) for functional assays, gene expression and morphology evaluation.

Human liver slices (HL870, HL871) were exposed to acetaminophen (APAP 1 mM) or diclofenac (DCF 1 mM) or the vehicle DMSO (0.1%) for 3 days. Dosing of drug treatment was achieved by replacement of the medium containing the drug at time 0, 24 and 48 h. At the time points, six treated human liver slices and 10 vehicle control slices were collected for functional assays and for gene expression.

### 4.4. Functional Assays

For ATP analysis, each slice was weighed, homogenized in 1.0 mL 10% trichloroacetic acid (TCA) and spun at 10,000 × g for 5 min at 4 °C. Homogenate supernatant samples (5 µL), diluted with 1 mL HEPES (25 mM), and added to a 96-well microtiter plate (200 µL). Standards were prepared in 10% TCA, diluted with HEPES, and 200 µL added per well in duplicate. The standard curve was from 0–200 µM ATP. The Luciferin-Luciferase solution (Luciferase 2 µg/mL, Luciferin 50 µM (5%), stabilizing buffer (50%), 25 mM HEPES buffer (45%)) was injected at 100 µL/well and luminescence was read using a Tropix TR717 Microplate Luminometer (Applied Biosystems, Bedford, MA, USA). Data for the liver or kidney slices are presented as nmoles/mg wet weight.

GSH content was determined on slice homogenate supernatants (50 µL), following a 10,000 × g centrifugation for 5 min at 4 °C, and transferred to a 96-well microtiter plate. Ellman’s reagent (200 µL; 39.6 mg dithiobis-nitrobenzoic acid/10 mL ethanol (EtOH) diluted 1:10 with 0.5 M Tris-1 mM EDTA buffer, pH 8.9) was added. The absorbance was determined at 405 nm using a Titertek Multiskan MCC/340 (Fisher Scientific, St. Louis, MO, USA). The values were compared to a standard curve of reduced glutathione (0–250 µM), and the data is presented as nmoles GSH/mg slice wet weight for the liver slices. Standards were prepared and added at 50 µL/well in duplicate.

K^+^ retention was determined by sonicating each slice in 250 µL distilled water and centrifuged (10,000 × g for 5 min). The resulting supernatant was analyzed for K^+^ content using a NOVA 8 electrolyte analyzer (Nova Biomedical, Boston, MA, USA). Results are expressed as µmoles K^+^/g slice wet weight.

For each of the functional assays, a one-way ANOVA followed by a Dunnett’s post-test was performed comparing the treated versus time-matched control values using GraphPad Prism software version 5.0 (GraphPad Software Inc., San Diego, CA, USA).

### 4.5. Morphology

The human liver or kidney slices were fixed flat in 10% neutral buffered formalin after transfer from the nitrocellulose insert to a 6-well culture dish via the incubation medium. The slices were stored refrigerated in the formalin for 24 h, placed between foam inserts in histological cassettes, and stored in 70% ethanol at 4 °C until paraffin embedding. Slices were cut, at 5 μM thickness, and processed for routine hematoxylin-eosin staining.

### 4.6. Gene Arrays

Affymetrix gene chip analysis was performed on slices from one human liver (HL714, four slices/time point, days 2–4) and one human kidney (HK1, four slices/time point, days 2–6) compared to day 1. Each organ was selected from five livers and five kidneys exhibiting the best viability based on the intracellular K^+^ values at the acquisition of the organs along with ATP and GSH slice levels during the culture period. The RNA processing and array analysis of the human liver slices is described previously, and the human kidney slices were processed the same as the liver [26]. In brief, total RNA was isolated from disrupted slices in RNA lysis buffer (guanidinium isothiocyanate and 1% β-mercaptoethanol) and processed using the Qiagen RNeasy 96 plate method. RNA yield and purity exhibited an OD_260/280_ ratio of ~1.7–2.0 which signified an RNA sample pure enough for use in expression analysis. RNA integrity (18s and 28s rRNA) was verified using the Agilent 2100 Bioanalyzer.

Double stranded cDNA was generated from 5–10 µg of total RNA/slice using the Superscript Choice System (Life Technologies, Rockville, MD, USA). The cDNA was purified by phenol/chlorophorm extraction and ethanol precipitation, and then transcribed to form biotin labeled cRNA using Enzo BioArray^®^ High Yield RNA Transcript Labeling Kit (ENZO, Farmingdale, NY, USA). The cRNA (20 µg cRNA; ≥ 0.6 µg/µL) was fragmented and hybridized (12–15 µg cRNA, ≥ 0.05 µg/µL) to U133A human genome arrays (Affymetrix, Santa Clara, CA, USA) for 16 h at 45 °C. The arrays were washed and stained using the GeneChip Fluidics station and scanned twice with the Gene Array^®^ scanner (Affymetrix). The hybridization intensity (.DAT file) was processed using the Affymetrix GeneChip Laboratory Information Management System (LIMS, Affymetrix, Santa Clara, CA, USA) and the average intensities for all probes cells (.CEL file) were converted to expression levels using a target intensity of 150. Statistical analysis was performed using PartekPro Software version 5.1 (St. Charles, MO, USA). Statistical significant genes were designated as those with a *p* value < 0.01 based on the analysis of variance and a randomization experiments to correct for multiple comparisons.

### 4.7. PCR Arrays

In this study, human specific qRT-PCR arrays of focused sets of genes, the Human Wound Healing RT^2^ Profiler (PAHS-121Z, 84 genes) and the Human Molecular Toxicology Pathway Finder RT^2^ Profiler (PAHS-3401, 370 genes) were processed on human liver slices from two livers (HL870, HL871) at Qiagen Sciences (Frederick, MD, USA). Total RNA was isolated from 10 control slices and six treated slices/drug/time point using Qiagen RNeasy mini kits with a trizol chloroform extraction by Qiagen Sciences, Inc. (Frederick, MD, USA). RNA samples were visualized and assayed for quality using the Agilent 2100 Bioanalyzer (Agilent Technologies, Inc., Foster City, CA, USA). RNA used for amplification had an RNA integrity number (RIN) ≥ 6.0 using the NanoDrop spectrophotometer (Thermo Scientific, Inc., Waltham, MA, USA). Cycle threshold (Ct) values of each sample were obtained and normalized to Ct values of the housekeeping genes (Actb, B2M, GAPDH, Hprt-1, Rplp-0). For statistical analysis gene ranking, which is comprised of fold change, and its *p*-value was calculated into one meta-characteristic; the algorithm is described elsewhere [48]. Fold change was based on a ratio of average log_2_ intensities of each gene in drug treated and vehicle treated liver slices, and a two-tailed Student’s *t*-test (*p*-value ≤ 0.05) to estimate significance between gene expression fold-change in drug treated and vehicle treated slices. Additionally, changes with a false discovery rate (FDR) of 15% or 30% were identified.

## 5. Conclusions

Human liver slices exhibit repair and regeneration, as well as provide insight into drug-induced injury underlying clinical changes. Initially, untreated human liver slices and human kidney slices maintained in roller cultures with the medium changed daily exhibited gene expression profiles indicative of tissue repair. Morphologically, the human liver slices exhibited regeneration with a variation in the size of the nuclei (anisokaryosis) and scattered multinucleated hepatocytes; the human kidney slices displayed evidence of progressive necrosis. Then, human liver slices were exposed to daily doses of APAP and DCF to stress the coping mechanisms induced by oxidative stress and to provide insight into the liver dysfunction associated with both drugs. Gene expression changes included oxidative stress, mitochondria energy, heat shock and ER stress, which were accompanied by altered ATP and GSH tissue levels. Additionally, wound repair genes were suppressed by DCF to a greater extent than APAP.

## Figures and Tables

**Table 1:** Summary of expression levels of genes altered significantly in untreated human liver and kidney slices maintained in culture with the daily exchange of medium. Gene expression changes were detected using the human U133A Affymetrix genome array for days 2–4 and compared to day 1 from human liver slices (HL714, four slices/time point) and human kidney slices (HK3, four slices/time point).

Gene Category	Human Liver			Human Kidney		
	Day 2	Day 3	Day 4	Day 2	Day 3	Day 4
**Extracellular Matrix**						
*Collagen type IV α1*	2.3	3.4	4.6			
*Collagen type IV α2*	1.9	3.3	4.0			
*Collagen type V α1*	2.0	2.7	3.7	1.1	1.1	1.8
*Collagen type V α2*	2.1	3.4	4.9	1.2	1.9	3.7
*Collagen type VI α3*	2.6	5.8	7.3	1.8	2.6	4.0
*Collagen type XV α1*	3.8	8.3	9.9	1.9	2.5	4.0
*Laminin β1*	1.6	2.4	2.9			
*Laminin γ1*	1.6	1.4	2.5			
*Collagen catabolism*						
*Matrix Metalloproteinase 1*				6.8	14.0	19.4
*Matrix Metalloproteinase 2*				1.4	1.6	2.4
*Matrix Metalloproteinase 3*				1.6	2.6	3.6
*Matrix Metalloproteinase 7*	1.6	4.5	5.7	1.1	1.8	2.2
*Matrix Metalloproteinase 12*				1.6	1.8	4.9
*Matrix metalloproteinase 14*				1.5	1.5	2.0
*TIMP-1*				1.2	1.3	1.5
*Renin*				1.1	1.8	3.5
**Cell Growth, Cell Cycle**						
*EGF*	2.4	2.6	3.0			
*Epiregulin*	11.1	8.4	6.8			
*TGF-β*	1.8	2.3	3.9	1.3	1.5	2.0
*Cell division cycle 2*				4.9	21.6	18.2
*Cyclin A2*				3.1	8.2	6.0
*Cyclin B2*				1.6	4.6	5.7
*MCM4*				2.9	3.1	2.5
*MCM5*				6.5	4.9	5.2
*NIMA*				4.8	23.8	25.7
*Kinesin family member 11*				3.6	5.1	4.7
*Kinesin family member 14*				1.9	4.5	3.7
*Polymerase, epsilon gamma*				2.6	3.1	3.1
*Proliferating cell nuclear antigen*	1.6	2.3	2.1	2.0	1.8	1.7
**Signal Transduction**						
*IL-1β*				1.4	1.5	1.8
*IL-24*				3.6	4.5	10.5

**Table 2:** List of genes significantly altered in expression by exposure to APAP (1 mM) and DCF (1 mM) for 72 h in human liver slices (HL870, HL871) following analysis on the Human Molecular Toxicology PathwayFinder RT^2^ PCR array (10 control and six treated slices/liver). Values represent the statistical analysis of gene ranking, which includes FDR adjusted p-values with thresholds of 15% and 30%.

Genes	APAP (1 mM)				DCF (1 mM)	
	HL870	HL871	HL870	HL871	HL870	HL871
	FDR < 15%	FDR < 15%	FDR < 30%	FDR < 30%	FDR < 30%	FDR < 30%
**Phospholipidosis**						
*ALDH1A1*	2.2					
*ASNS*					7.1	7.1
*CES2*						−3.3
*CTSB*					−4.3	−5.5
*FABP1*				−1.8	−25.2	−54.7
*GSTM4*					−3.6	−4.2
*HPN*						−3.6
*INHBE*	−4.7	−6.4			−4.6	
*NR0B2*						−5.2
*S100A8*	−2.8	−2.9				
*SERPINA3*				−1.5		
*TAGLN*					−6.9	
*TIMM10B*					5.3	
*WIPI1*					3.6	4.0
**Steatosis**						
*ACACA*		−2.1				
*ADK*					3.5	
*ALDH1A1*	2.2					−6.7
*DNM1*				−2.0		
*FASN*		−2.7	−1.5			−19.3
*GPD1*					−5.7	
*KHK*					−3.2	−6.4
*LPL*			1.8			
*MAPK8*			1.2		4.6	
*MTTP*					−14.1	−17.7
*PNPLA3*		−2.0				
*SCD*	−2.7	−2.5			−11.1	−14.2
*SREBF1*	−2.0	−2.9			−5.5	−7.4
**Cholestasis**						
*ABCB1*			1.8		3.0	
*ABCB4*		−2.3				
*ABCC1*				−1.8		
*ABCC2*	2.9	2.0				
*ABCC3*			1.5			
*APOA5*				−1.8		
*APOE*			−1.6			
*APOF*	−2.5	−1.9			−6.7	−12.2
*CYP7A1*	4.5	3.3				−23.1
*CYP7B1*					−8.9	−8.2
*ESR1*			1.9			
*HLA−DRB1*					−12.8	
*JAG1*				−1.5		
*NR1H4*			−1.4		−4.2	−4.9
*NUP210*					−7.0	
*PDYN*				−2.1		
*SLC10A*						−8.3
*SLC51A*					−2.8	−8.8
*SLCO1A2*					−19.2	−54.6
*UGT1A1*					−5.6	−8.8
*UGT2B4*	3.2	2.7				
**Fatty Acid Metabolism**						
*ACAA1*			1.4			
*ACAD11*			1.5			
*ACADSB*						−6.2
*ACAT1*			−1.5	−1.7		
*ACAT2*						−20.7
*ACOT1*				−1.7		
*ACOX2*						−20.7
*ADH1C*			3.2			−30.6
*CPT1B*			−1.6			
*CPT2*			1.9	−1.7		
*ECHS1*						−4.1
*EHHADH*						−5.0
*GCDH*	1.6					
**Oxidative Stress**						
*CAT*						−3.5
*DHCR24*						−5.6
*DUOX1*	−2.8	−2.9			−45.5	
*DUOX2*	−2.6			−2.3	−16.2	
*EPHX1*	2.6					−7.6
*GPX1*					1.9	
*GPX2*	1.5				−31.2	−24.6
*GPX4*	1.6				2.2	
*GPX7*			−1.5	−1.5		
*GSTA3*	2.5					
*MPO*		−2.6				
*NQO1*	1.7	1.7				
*POR*			1.4		4.1	2.8
*PPP1R15B*					4.5	3.7
*PRDX1*			1.5			
*PRDX2*			1.3			
*PRDX6*	1.4	1.5			3.0	
*TXNIP*			−1.5	−1.8	−9.1	
**Mitochondria Energy**						
*ACLY*		−1.6				
*COX6B1*					1.9	
*CYC1*			1.4			
*IDH1*			1.5			−5.3
*IDH2*		−2.0				
*IDH3G*			1.3			
*MDH1B*			−2.4	−2.4		
*SDHA*			1.5			
*SDHC*						−3.4
*SUCLG1*				3.0		
*UCP2*					−7.5	−6.0
**Heat Shock**						
*CRYAA*		−5.0				
*DNAJA2*					2.5	
*DNAJB1*					3.1	
*DNAJB6*					2.4	
*DNAJC3*				−1.5		
*HSF1*			1.6			
*HSF2*					4.1	4.2
*HSP90AA1*			1.5	1.3		
*HSPA1A*	1.9					
*HSPA1B*	2.1					
*HSPA9*					3.1	2.8
*HSPBAP1*	−1.6					
*HSPB6*			−1.5	−1.6		
**ER Stress**						
*AMFR*				−1.4		
*ATF4*					5.6	4.6
*ATF6*					4.4	2.9
*DDIT3*			−1.5			
*DERL1*					4.7	
*EDEM1*				−1.5		
*EIF2AK3*				−1.7	2.9	3.6
*ERO1LB*					3.5	
*FBXO6*					2.7	
*HTRA4*			−2.3			
*SERP1*				−1.5	2.7	
*VCP*			1.5			
*VIMP*					4.5	
*XBP1*				−1.6		
**Metabolism**						
*CYP1A2*					−47.1	−246.2
*CYP2B6*	4.4			1.8		−19.9
*CYP2C9*				2.6		
*CPY2C19*			2.0			−33.7
*CYP2D6*			−1.4			−4.0
*CYP2E1*			−1.8			
*CYP3A4*	3.2					
*GSTA3*					−14.5	−77.1
*FMO2*					−8.4	
*FMO3*						−5.8
*FMO4*					−5.6	−4.7
*FMO5*						−19.9
*UGT2B4*					−4.4	−9.4
**DNA damage/repair**						
*CDKN1A*			−1.4			
*DDIT3(GADD153)*					13.8	17.0
*ERCC1*					3.8	4.3
*ERCC5*					2.3	
*MDM2*					4.1	
*MKI67*						−52.1
*PCNA*			1.6			
*XRCC1*					2.5	
**Apoptosis**						
*AKT1*				−1.5		
*BAD*				−1.5		
*BAK1*						3.2
*BCL2*	−1.6					
*BCL2L11*		−2.3	−1.4			
*CASP1*					−8.0	
*FASLG*			−1.8			
*GADD45A*					23.4	22.9
*MCL1*			−1.3			
*TNFSF10A*				−1.3	2.8	1.9
*TNFRSF10B*					5.2	3.3
*TP53*					2.7	
**Necrosis**						
*ATP6V1G2*				−2.4		
*BMF*	−2.4	−2.0				
*CCDC103*		−3.0				
*CLEC18A*				−2.6		
*CYLD*					3.9	4.0
*GALNT5*				−2.5		
*HSPBAP1*				−1.7		
*MAG*			1.4	−1.6		
*PARP2*					3.1	2.9
*RAB25*			−1.8	−3.8	−6.3	
*SPATA2*			2.1		6.3	
*TNFAIP8L1*					−7.1	−11.0
*TXNL4B*					3.2	2.8
**Inflammation/Immunotoxicity**					
*AHSG*			−1.5		−4.1	
*C3*		−3.0			−12.4	−16.0
*C9*		−1.7	−1.6		−14.9	−15.2
*CD4*						−36.7
*CD36*					−25.8	−23.3
*CD80*					−29.1	
*CD86*			−1.7	−1.6	−62.4	−9.5
*CTSE*	−2.4				−17.8	
*F2*			−1.4			
*HRG*	3.0				−9.6	−56.1
*IL1B*					−43.7	−16.6
*IL4*				−2.1		
*IL6*	−2.3	−2.4				
*IL10*			−1.8			
*ITGAX*					−24.0	−12.3
*KLF1*					4.0	
*LYZ*	−1.7			−1.5		−20.1
*METAP2*					1.8	
*PON1*						−8.2
*POU3F3*			1.4			
*PTPRC*			−1.6			
*TNF*		2.1			−10.1	

**Table 3:** Percentage of genes significantly altered by APAP (1 mM) and DCF (1 mM) exposure at 48 and 72 h in human liver slices (HL870, HL871) from the Human Molecular Toxicology Pathway Finder RT^2^ PCR array.

Gene Categories	APAP (1 mM) % Genes/Category				DCF (1 mM) % Genes/Category			
	HL870 48 h 72 h		HL871 48 h 72 h		HL870 48 h 72 h		HL871 48 h 72 h	
Cholestasis, Steatosis, Phospholipidosis	29.0	27.3	19.6	33.3	20.9	29.2	19.6	33.8
Fatty acid Metabolism	9.7	9.1	6.5	4.8	7.0	1.1	5.9	8.1
Oxidative Stress	12.9	14.3	8.7	11.1	9.3	9.0	5.9	5.4
Mitochondria Energy	9.7	6.5	4.3	5.2	2.3	2.3	2.0	4.1
Heat Shock, ER Stress	12.9	11.7	15.2	14.3	25.6	18.0	27.5	8.1
Metabolism	9.7	6.5	6.5	3.2	7.0	6.7	9.8	13.5
DNA Damage/Repair	0	2.6	4.4	0	4.7	6.7	3.9	2.7
Apoptosis	3.2	5.2	10.9	6.4	14.0	5.6	15.7	6.8
Necrosis	6.5	5.2	15.2	12.7	0	6.7	2.0	5.4
Inflammation/Immunotoxicity	6.5	11.7	8.7	7.9	9.3	14.6	7.8	12.2
Number of significant gene changes	31	77	46	63	43	89	51	74

**Table 4:** List of genes significantly altered in expression by exposure to APAP (1 mM) and DCF (1 mM) for 72 h in human liver (HL870, HL871) slices (10 control and six treated slices/liver) from the Human Wound Healing RT^2^ PCR array. Values represent the statistical analysis of gene ranking, which includes FDR adjusted *p*-values with thresholds of 15% and 30%.

Genes	APAP (1 mM)			DCF (1 mM)		
	HL871	HL870	HL871	HL871	HL870	HL871
	FDR < 15%	FDR < 30%	FDR < 30%	FDR < 15%	FDR < 30%	FDR < 30%
**ECM Structural**						
*COL1A1*	−2.4			−73.9	−38.3	
*COL1A2*		−1.6		−20.1	−178.8	
*COL3A1*		−1.8	−1.8	−22.8	−140.2	
*COL5A2*		−1.4	−1.8		−8.2	
*COL5A3*	−2.9	−2.2				
*VTN*					−2.2	−3.3
**ECM Remodeling**						
*FGA*					−12.9	−6.4
*MMP1*				12.8	25.1	
*MMP7*		−1.6	−1.9	−56.4	−1.3	
*MMP9*					−17.3	
*PLAU*		−1.7			−11.4	
*PLAUR*		−1.7				−4.5
*PLG*						−10.5
*SERPINE1*		−1.5				
*TIMP1*						−4.0
**Cell Adhesion**						
*ITGA1*	−2.1	−1.6				
*ITGA2*	−1.8	−1.4				
*ITGA3*			−1.8			
*ITGA4*				−20.8	−26.2	
*ITGA5*	−1.9	−1.7			−3.6	−3.5
*ITGB6*		−3.1				
*TAGLN*					−11.2	−4.2
**Inflammation, Cytokines**						
*CCL2*					−22.7	
*CXCL1*		−1.8				
*CXCL2*						−5.7
*CXCL5*		−2.0			−38.7	−12.2
*CXCL11*			2.2			
*IFNG*		2.3				
*IL1B*					−56.0	−21.7
*IL6*	−3.6	−1.9				
**Growth Factors**						
*CSF3*					53.9	50.3
*EGF*		2.1				
*FGF2*					9.0	5.3
*FGF7*		−1.7			−13.5	
*HBEGF*					4.5	
*HGF*		−1.6				
*PDGA*					3.8	
*TGFB1*						−4.7
*TNF*			1.8			−10.3
*VEGFA*		−1.6	−1.6			
**Signaling**						
*PTGS2*		−1.7	−2.2	25.4	29.0	
*WISP1*					−15.2	−11.1

**Table 5:** Human liver and kidney donor information, plus markers of viability and tissue quality (K^+^, ATP and GSH slice levels) of control slices (24 h).

Donor	Age/Sex/Race	Cold Ischemia (h)	K^+^ μmols/g Wet Weight 24 h	ATP nmols/mg Wet Weight 24 h	GSH nmols/mg Wet Weight 24 h
HL714	M/C/46	12	75.9	16.8	5.6
HK3	F/C/49	12.5	85.0	8.3	21.8
HL870	M/C/21	14	80.4	15.0	10.6
HL871	F/A/28	16	78.9	13.5	16.5

HL, human liver; HK, human kidney; M, male; F, female: C, Caucasian; A, Asian.
